# Global maxillary ridge augmentation with frozen radiation-sterilised bone blocks followed by implant placement: a case report

**DOI:** 10.1007/s10561-014-9452-y

**Published:** 2014-05-13

**Authors:** Marta Krasny, Kornel Krasny, Artur Kamiński, Piotr Fiedor

**Affiliations:** 1 Medicare Dental Practice, Warsaw, Poland; 2 Department of Orthodontics, Medical University of Warsaw, Warsaw, Poland; 3 Department of Transplantology and Central Tissue Bank, Medical University of Warsaw, Warsaw, Poland; 4 Department of General and Transplantation Surgery, Transplantation Institute, Medical University of Warsaw, Warsaw, Poland; 5 ul. Cicha 43, 05-074 Halinów, Poland

**Keywords:** Allografts, Preimplantation preparation, Atrophic alveolar ridge reconstruction

## Abstract

Due to atrophy of the tissue within the alveolar ridge, implantation must sometimes be preceded by bone regeneration. The use of allogeneic material allows the surgeon to prepare grafts of any shape and amount; therefore it is a good alternative to autograft reconstruction in patients with extensive atrophy of the alveolar ridge. The patient with maxillary anodontia showed insufficient width of the ridge along its entire length, which prevented implantation. Therefore, alveolar ridge reconstruction was planned. Four frozen, radiation-sterilised bone blocks processed in the Tissue Bank in Warsaw were used for reconstruction of the alveolar ridge. The blocks were grafted to the area of molars, premolars and lateral incisors bilaterally. Three months after surgery a normal union of transplants with the recipient site was achieved. Six implants were embedded and following the 6-month integration period a permanent prosthetic restoration was successfully performed. During a 38-month follow-up none of the implants were lost and the aesthetic or functional condition of the prosthetic restoration did not deteriorate. Frozen allogeneic radiation-sterilised bone blocks constitute good, efficient and safe material used in reconstruction of the alveolar ridge in extensive bone atrophy. This is only one of possible grafting materials for reconstruction of extremely atrophic alveolar ridge.

## Introduction

Post-extraction bone tissue atrophy in the alveolar ridge was found both, in vertical and horizontal dimensions (Chackartchi and Stabholz 2013). The systematic reviews demonstrated that the alveolar ridge underwent mean horizontal reduction in width of 3.8 mm and mean vertical reduction in height of 1.24 mm within this time (Hämmerle et al. 2011). The bone loss in the maxilla was more significant than in the mandible and in both cases atrophy on the vestibular side prevailed, which is associated with its reduced bone width within this location (Irinakis 2006). The process occurs mostly during the first 5–6 weeks following tooth extraction (Amler 1993) but a complete rebuilding process lasts approximately 6 months. Combined with traumatic extraction or chronic inflammations of the periodontium surrounding teeth qualified for extraction, the dimensions of the remnant alveolar ridge may be too small to allow implantation.

Reconstruction of the alveolar ridge most often is performed through autografting or alternatively by the use of allografts or bone replacement materials (Saravanan et al. 2013; Krasny et al. 2013; Pierrefeu et al. 2012). In extensive atrophy of the alveolar ridge three treatment modalities are most commonly chosen. Autografting from the ilium constitutes a burden for the patient, requires general anaesthesia and subjects them to additional complications related to the donor site (Barone and Covani 2007). Bone distraction requires additional surgical procedure as well as time for recovery (Zwetyenga 2012). The third solution is the use of allogeneic bone blocks, the availability of which combined with their nearly unlimited dimensions, easy processing and adjusting to the recipient site gathers an increasing number of followers (Wallace et al. 2013). Although allografts with unlimited size seem to be the best option, the risk of infection transmission from the donor to the recipient has to be taken into account (Eastlund 2006). Therefore, donor screening based on medical and social history, donor physical examination and autopsy results (if applicable), as well as biological examination of blood, are required for proper evaluation of donors (Pruss et al. 2010). Additionally the introduction of secondary sterilisation reduces the abovementioned risk related to tissue allografts (Loty et al. 1990). However, it was shown, that the use of irradiation as a sterilisation method may impair bone allograft properties (Cornu et al. 2000).

## Patient and methods

**Fig. 1:**
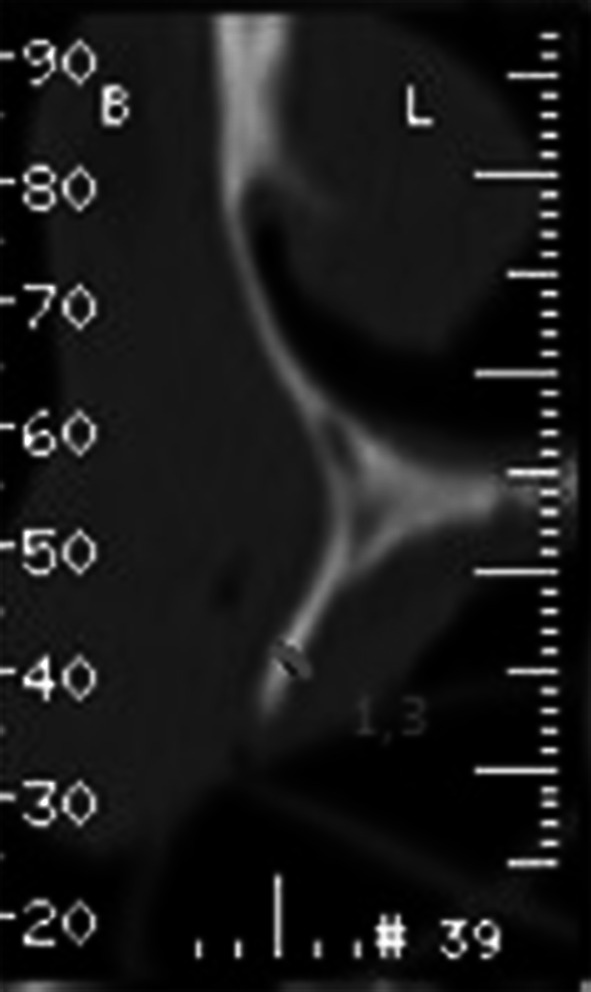
CT before grafting within location of tooth 16

A 56-year old patient presented himself for prosthetic rehabilitation of the edentulous maxilla. The patient reported that the last teeth were extracted several years before and he used a complete removable prosthesis which he did not approve. The patient reported enormous discomfort due to insufficient retention of the prosthesis and unsatisfactory adhesion to the gingiva as well as taste disturbances. The intraoral examination revealed a narrow, atrophic alveolar ridge and high palatal vault (gothic palatal arch). Computed tomography imaging showed extensive bone loss throughout the entire maxillary alveolar ridge (Fig. 1).

The following four treatment modalities were discussed:

1. Complete acrylic prosthesis supported by mucous membrane.
2. Complete acrylic prosthesis supported by two, three or four implants.
3. Mixed prosthesis consisting of an implant-based fixed prosthesis in the front (3–3) and removable prosthesis supported by crowns fixed on implants in lateral segments.
4. Fixed prosthesis supported by six implants.

In view of previous experience of the patient, modalities, which involved a removable prosthesis, were excluded. Moreover, the patient wished for a fixed prosthesis, which would not have to be removed from his mouth. Therefore, a fixed prosthesis supported by six implants was chosen. The implant-prosthetic plan provided for restoration of 12 teeth (16–26).

The required bone graft involving the entire length of the alveolar ridge due to its considerable atrophy was discussed with the patient. Since the necessary amount of material exceeded the capacity of the intraoral donor site, autologous iliac bone grafting was considered, which was rejected by the patient. Another option was allogeneic material from the Tissue Bank. Allogeneic bone blocks were processed from iliac ala of deceased donors and subsequently radiation-sterilised with a dose of 35 kGy on dry ice using an Electron Beam Accelerator (LAE-10; 10 MeV). Bone allografts were stored frozen until distribution. The patient accepted a graft consisting of four independent bone blocks, filled in and signed required documents.

### Surgical procedure

**Fig. 2:**
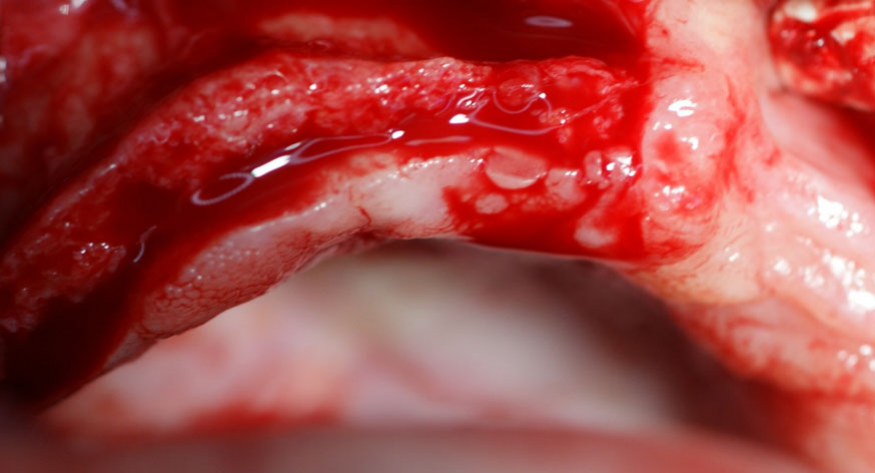
Narrow alveolar process

The procedure was performed under local anaesthesia with 4 % Ubistesin Forte. An incision in the mucous membrane was done beginning at teeth 17–11. The incision was then extended to the vault of the oral vestibule. The mucoperiosteal flap was detached. Reduced width of the maxillary alveolar ridge was confirmed intraoperatively (Fig. 2), which was consistent with prior CT finding. No gross pathological changes were found. The width of the ridge ranged from 1 to 2 mm and the height—from 8 to 15 mm along the entire ridge.

**Fig. 3:**
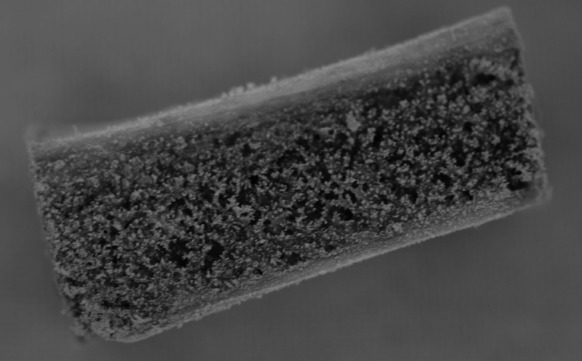
20 × 10 × 10 mm frozen, radiation-sterilised bone block

Based on clinical and radiological (CT) findings four bone blocks (compact-trabecular bone) were ordered from the Tissue Bank. Two of them were 20 × 10 × 10 mm and two were 10 × 10 × 10 mm (Fig. 3). The bone blocks were harvested from the iliac ala and radio-sterilised with a dose of 35 kGy.

**Fig. 4:**
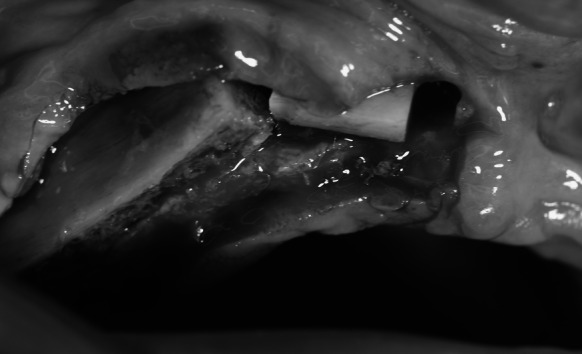
Right-side location of oral allografts

The bone block was aligned so the compact lamella was the external layer—located towards the vestibule and the trabecular layer of the block (internal layer) was shaped so it could precisely adhere to the recipient site in the maxilla and then the sharp bone edges were smoothed. Due to anatomic features the longer block was fixed within the area of teeth 16–14 and the smaller bone block was located within the area of tooth 12 having considered the curve of the alveolar arch. The final shape of graft was obtained intraorally. Each of the blocks was secured with two MEISINGER titanium screws (Fig. 4). Shavings formed as a result of shaping of the bone blocks were placed in the space between the donor and recipient sites as well as on each side of the bone block. The operational site was covered with platelet rich plasma membrane and with a free mucous membrane flap. The wound was sutured with 4.0 Safil HR 22 in a way preventing excessive stretching of the flap. On the left side bone grafting was performed in a similar manner.

A 7-day per os antibiotic therapy was prescribed to the patient as well as analgesics and antibacterial mouth rinse to follow the procedure. Cold packs were used to reduce postoperative oedema. No postoperative complications were observed. The sutures were removed 14 days after surgery. The patient was referred to monthly surgical follow-up. Due to considerable widening of the ridge a new complete upper prosthesis was made for temporary use, which did not apply any pressure to the bone grafting sites.

**Fig. 5:**
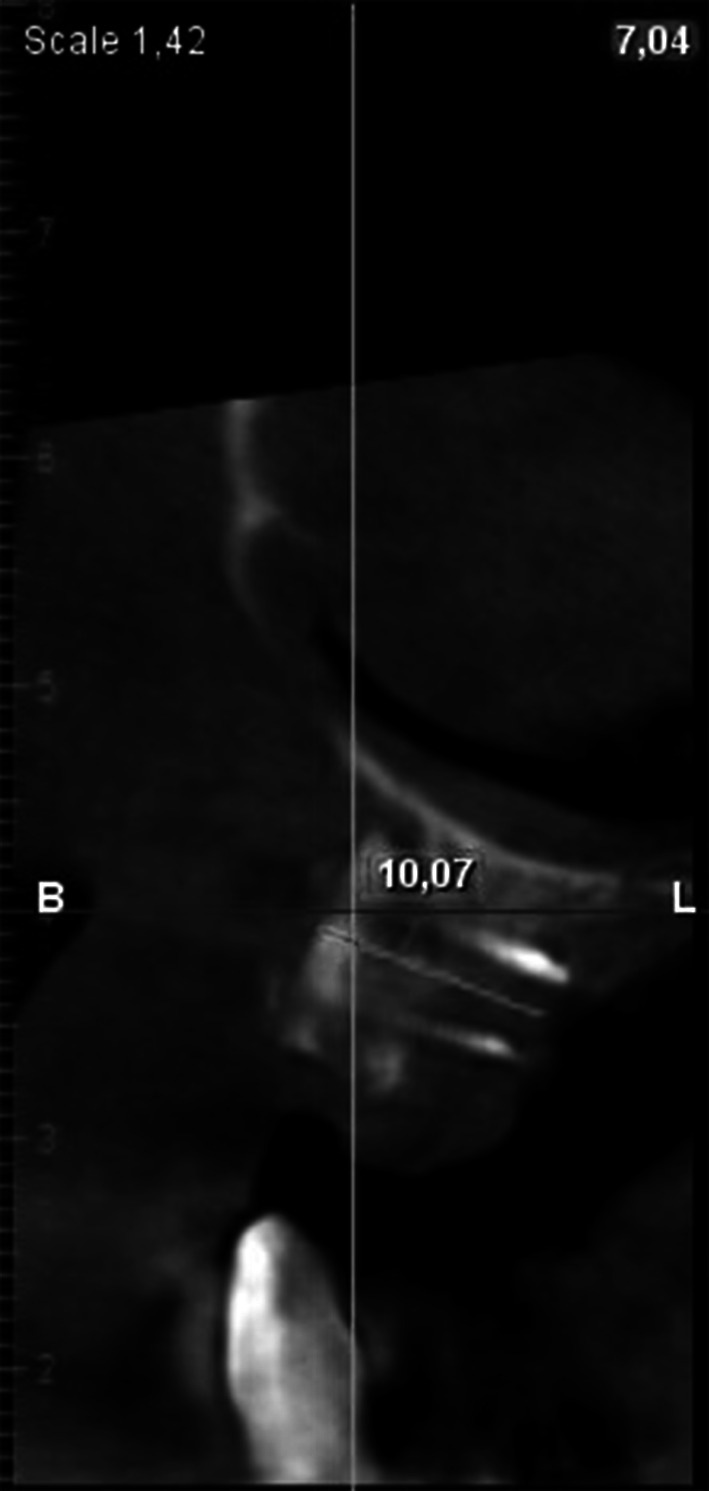
CT after grafting, the same location

**Fig. 6:**
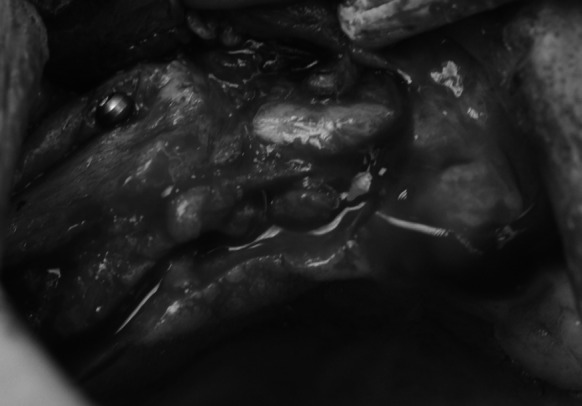
Healed bone block grafts; bone bed ready for implantation

A follow-up CT imaging (Fig. 5) was performed after 6 months. The result indicated normal bone union and slight bone atrophy at the margins of the grafts. The intraoral examination showed normal mucous membrane and a wide alveolar ridge of optimal height (Fig. 6).

**Fig. 7:**
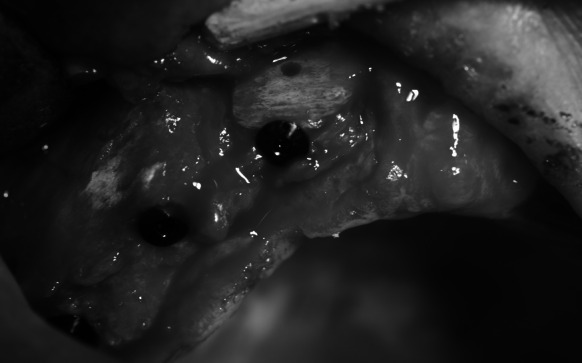
Implants embedded in the bone

The second part of the plan was commenced and under local anaesthesia with 4 % Ubistesin Forte six implants were embedded within the area of teeth 16, 14, 12, 22, 24, 26. After the mucoperiosteal flap was detached, normal union of the patient’s bone and the homogenic bone was found. The graft-securing screws were removed and six BIOMET 3I implants (16-NINT 485, 14-NINT 410, 12-NINT 3211, 22-NINT 3211, 24-NINT 3210, 26-NINT 3210) (Fig. 7) were embedded. During the procedure (the implants were embedded within the grafted area only) bleeding was observed, which indicated normal revascularisation of the graft. After normal primary stability was determined unequivocally, the closing screws were fixed and the wound was sutured.

Just as it was in case of the first procedure, the patient underwent 7-day antibiotic therapy and was recommended to use analgesics and antibacterial mouth rinse. No perioperative or postoperative complications were found. The sutures were removed after 14 days and at the same time the prosthesis was slightly corrected so it did not apply any pressure on implants.

### Restorative procedure

Following 6 months of osseointegration the implants were uncovered under local anaesthesia. After normal secondary stability was determined, healing screws were fixed for a period of 2 weeks. Later, at subsequent visits the height of occlusion was established and full arch bridge impressions were taken to restore the defects of teeth 16–26. Balanced occlusion was obtained following correction of contact points of opposing teeth. At the last visit a porcelain-fused-to-metal bridge was cemented, which completed the 13-month treatment period.

### Long term results

**Fig. 8:**
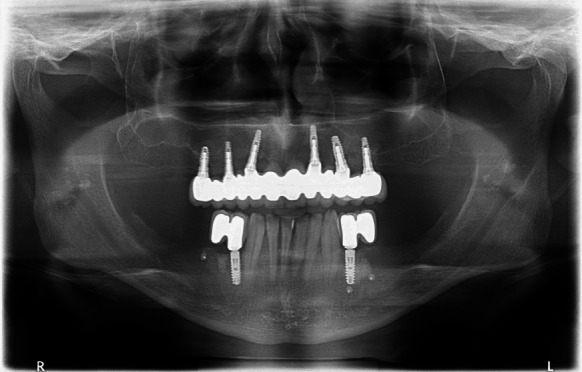
OPG after a 38-month follow up

During the 38-month follow-up period no case of a lost implant was found. Secondary stabilisation was maintained in all the grafts, the double porosity surface contributed to improved integration with the bone tissue under reconstruction. Clinical assessment and intraoral examination and OPG (Fig. 8) did not demonstrate gingival recession or bone atrophy around the implants, which confirmed the long-term follow-up efficacy and stability of the treatment.

## Discussion

Many scientists and dental practitioners as well as the authors of this case report, believe that the best grafting material is the autograft (Acocella et al. 2010; Hyeon-Jung et al. 2007; Draenert et al. 2013). It eliminates the risk of spreading infectious diseases such as HIV infection as well as prions between the donor and the recipient, there is no rejection reaction and the highest efficacy and predictability of the procedure are provided (Sutherland et al. 1997; Cook et al. 1995). This procedure was also considered in the discussed case. However, in view of the large amount of material required, the number of donor sites was limited. Moreover, considering the complications of an additional procedure of harvesting a bone graft from a donor site, such as hindered walking, delayed healing or the risk of the procedure itself, finally a frozen, radiation-sterilised, compact-trabecular bone allograft was chosen. The advantages of this decision were almost unlimited availability of grafted tissues as well as possibility to freely modify the size and shape of them (Kim et al. 2010). Allogeneic bone block provides a predictable reconstruction in both faciolingual and vertical directions (Schwartz-Arad et al. 2005).

Dispute ensued over whether such material may transfer diseases. There have been several reports published describing the transmission of bacterial (Eastlund 2006) and viral infections (Eastlund 2005), including human immunodeficiency virus type 1—HIV-1, hepatitis *C* virus (Conrad et al. 1995; CDC 2011) with bone allografts procured and processed under aseptic conditions and preserved by freezing. Moreover, there have been publications proving that durability of such bone grafts deteriorates over the preparation process and, above all, during radiation-sterilisation (Pelker et al. 1993; Cornu et al. 2000).

Other studies (Dziedzic-Goclawska et al. 2005; Kaminski et al. 2012a, b), as well as the presented case report, proved that the concerns are groundless. Frozen bone grafts radiation-sterilised even with as high dose as 35 kGy were entirely safe for the recipient’s health and its structure provided excellent scaffold for the new bone formed in the process of osteoinduction. This is only one of possible grafting materials for reconstruction of extremely atrophic alveolar ridge.

Due to the lack of the permission of the bioethics committee it was impossible to perform the bone biopsy at the area of implantation. Marked bleeding observed during implant embedment indicating revascularisation was the only unbiased proof of the graft incorporation and remodelling.

Authors’ own experience implied that the size of the grafted block should be only slightly bigger than necessary to embed implants, because bone atrophy was found only at the margin of the allograft and around the securing screws. The material may be ordered in any shape and size and adjusted to the form of the recipient site, which provides an advantage over autograft material and seems to possess comparable biological properties.

The authors would like to emphasise the need of obtaining normal stability of the graft, i.e. durable fixation with screws, sealing the donor/recipient site borderline with bone shavings as well as covering the operational site with PRF membranes, which are a concentrate of growth factors such as: three pro-inflammatory cytokines (IL-1beta, IL-6, and TNF-alpha), an anti-inflammatory cytokine (IL-4), and a key growth promoter of angiogenesis (VEGF) and hence accelerate reorganisation of the graft (Dohan et al. 2006). It is also important to cover the wound with an unstretched mucous membrane to avoid its abrasion and exposing the allograft to the oral environment.
